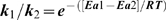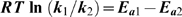# Functional Interactions between KCNE1 C-Terminus and the KCNQ1 Channel

**DOI:** 10.1371/journal.pone.0005143

**Published:** 2009-04-02

**Authors:** Jerri Chen, Renjian Zheng, Yonathan F. Melman, Thomas V. McDonald

**Affiliations:** 1 Department of Medicine, Albert Einstein College of Medicine, Bronx, New York, United States of America; 2 Department of Molecular Pharmacology, Albert Einstein College of Medicine, Bronx, New York, United States of America; 3 Department of Medicine, Brigham and Women's Hospital, Boston, Massachusetts, United States of America; University of Virginia, United States of America

## Abstract

The KCNE1 gene product (minK protein) associates with the cardiac KvLQT1 potassium channel (encoded by KCNQ1) to create the cardiac slowly activating delayed rectifier, I_Ks_. Mutations throughout both genes are linked to the hereditary cardiac arrhythmias in the Long QT Syndrome (LQTS). KCNE1 exerts its specific regulation of KCNQ1 activation via interactions between membrane-spanning segments of the two proteins. Less detailed attention has been focused on the role of the KCNE1 C-terminus in regulating channel behavior. We analyzed the effects of an LQT5 point mutation (D76N) and the truncation of the entire C-terminus (Δ70) on channel regulation, assembly and interaction. Both mutations significantly shifted voltage dependence of activation in the depolarizing direction and decreased I_Ks_ current density. They also accelerated rates of channel deactivation but notably, did not affect activation kinetics. Truncation of the C-terminus reduced the apparent affinity of KCNE1 for KCNQ1, resulting in impaired channel formation and presentation of KCNQ1/KCNE1 complexes to the surface. Complete saturation of KCNQ1 channels with KCNE1-Δ70 could be achieved by relative over-expression of the KCNE subunit. Rate-dependent facilitation of K^+^ conductance, a key property of I_Ks_ that enables action potential shortening at higher heart rates, was defective for both KCNE1 C-terminal mutations, and may contribute to the clinical phenotype of arrhythmias triggered by heart rate elevations during exercise in LQTS mutations. These results support several roles for KCNE1 C-terminus interaction with KCNQ1: regulation of channel assembly, open-state destabilization, and kinetics of channel deactivation.

## Introduction

The slowly activating delayed rectifier potassium current (I_Ks_) plays an important role in controlling the repolarizing phase of the cardiac action potential, particularly during periods of elevated heart rate. I_Ks_ is carried by channels comprised of KCNQ1 pore-forming subunits and KCNE1 regulatory subunits [1], [2]. The KCNE1 gene product (minK) is a small protein of 129 amino acids that belongs to the family of K^+^ channel-interacting KCNE proteins, the members of which share structural similarities: a single membrane-spanning domain, and short C- and N- terminal tails (intracellular and extracellular, respectively) [3], [4]. Though KCNQ1 expression alone generates a K^+^ current, association with KCNE1 is required for the current to recapitulate cardiac I_Ks_, with its slow activation and shifted voltage-dependence of activation [1], [2].

In addition to KCNE1, the other four members of the KCNE family (KCNE2-5, encoding proteins MiRP1-4, respectively) are capable of associating with KCNQ1 and regulating channel behavior [5]–[10]. Since each KCNE affects KCNQ1 channel gating differently, mutagenesis and chimeras have enabled investigators to probe which portions of the accessory subunits provide functional interactions and specificity. By this means, the structural determinants of KCNE1 and KCNE3 regulation of KCNQ1 have been investigated to identify the site that controls activation gating within the KCNE transmembrane domain (TMD) with single amino acid resolution [11], 12. The KCNQ1 S6 TMD has been analyzed by mutagenesis to identify those residues that interact with KNCE1 and KCNE3 and which differentially stabilize open or closed states to account for the widely differing kinetics of channel activation [13], [14].

While many studies have concentrated on the membrane-spanning region of KCNE1, the role of the cytoplasmic C-terminus has been less thoroughly explored. Several naturally occurring Long QT Syndrome (LQTS) mutations have been found in the C-terminus of KCNE1, as well as in the C-terminal tail of KCNQ1 that implicate the importance of these regions in the regulation of I_Ks_ [15]–[18] (http://www.fsm.it/cardmoc/). Additional mutations in KCNQ1 and other KCNE genes have been associated with familial atrial fibrillation [19]–[21]. Congenital mutations in KCNQ1 and KCNE1 (corresponding to LQTS subtypes LQT1 and LQT5, respectively), account for over 50% of inherited LQTS [22], and are associated with arrhythmias triggered by exercise-related increase in heart rate and β-adrenergic stimulation [23]. These findings underscore the ability of I_Ks_ to contribute to rate-related adaptation of cardiac repolarization and maintenance of normal sinus rhythm and excitability during stress.

In the present study, we focused our investigation on the role of the KCNE1 C-terminus on regulation and rate-adaptation of I_Ks_. Our data show that deactivation kinetics—a parameter that has thus far been given little attention—is significantly affected by mutations in the C-terminus. We also provide evidence that the C-terminus truncation or mutation also renders the channel incapable of adapting I_Ks_ current accumulation in response to increases in pulse rate as does the channel formed by wild-type KCNE1. Thus, interactions between the KCNE1 C-terminus and KCNQ1 may play a critical physiological role in rate-related adaptation of action potential duration, the dysregulation of which could contribute to LQT1 and LQT5 phenotypes.

## Results

**Figure 1:**
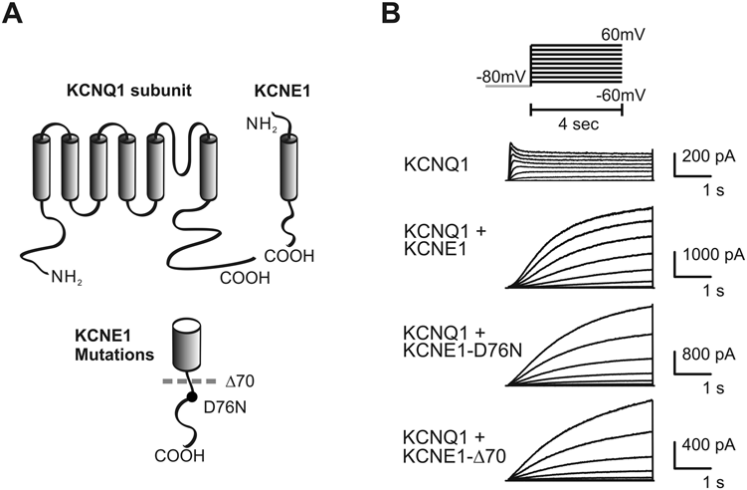
A) Schematic of KCNQ1 subunit and KCNE1, showing location of KCNE1 mutations. B) Current traces of activation protocol, with protocol in inset.

**Figure 2:**
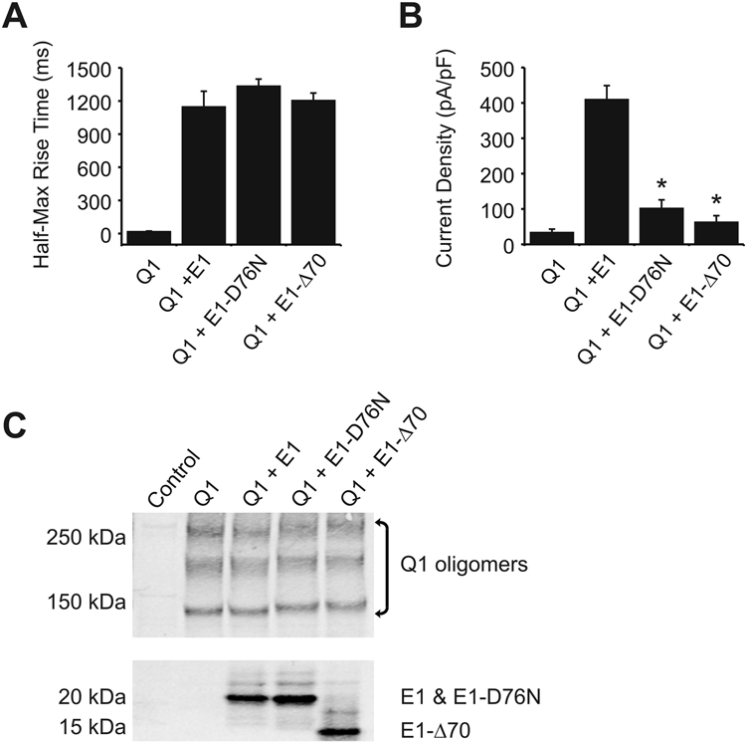
KCNE1 mutations D76N and Δ70 reduce I_Ks_ current density with minimal effects on activation kinetics. A) rates of activation, and B) current density from CHO cells transiently expressing Myc-tagged KCNQ1 (Q1) either without KCNE1 (E1), or with wild-type FLAG-tagged E1, E1-D76N, or E1-Δ70. Asterisks (*) indicate statistical significance to P<0.001. n = 6. C) Immunoblot showing KCNQ1 protein levels when expressed alone or co-expressed with KCNE1 (wild-type and mutant). Western blot analysis performed with mouse anti-Myc antibody (Santa Cruz) for KCNQ1, and mouse anti-FLAG antibody (Sigma) for KCNE1. Left lane in each gel shows signal from cells that are untransfected.

To examine the role of the cytoplasmic C-terminus of KCNE1 (amino acid residues 67–129) on KCNQ1/KCNE1 channel function, we examined two KCNE1 mutants: KCNE1-D76N, and KCNE1-Δ70 truncation mutant (Figure 1A). Wild-type KCNE1 and each mutant were transiently co-transfected with KCNQ1 in Chinese Hamster Ovary (CHO) cells, and current was assessed via whole-cell patch clamp. Compared to cells transfected with KCNQ1 alone, those transfected with KCNQ1 and wild-type KCNE1 (Figure 1B) exhibited the well-known transformed KCNQ1 current consistent with I_Ks_. Among the changes caused by KCNE1 are increase in current density, slowed activation/deactivation kinetics, shift in voltage dependence of activation (VDA) and abolishment of inactivation. The KCNE1 C-terminal mutations D76N and Δ70 also transformed KCNQ1 currents with minimal effects on activation rate. Since I_Ks_ does not completely saturate during activating depolarizing steps investigators have generally chosen to make comparative activation rate measurements in an isochronal fashion [24]. Furthermore, the current shows a sigmoidal time course of activation indicating multiple states prior to a conducting one, so for simplicity, the time to half maximal conductance has been accepted as a measure of relative activation rate [11], [13], [14], [25]. Figure 2A shows the summary data for activation rates, analyzed as the time required to reach half-maximal conductance. The current density in cells expressing channels comprised of KCNQ1/KCNE1-D76N were reduced compared to wild type KCNE1 (Figure 2B) and have been attributed to changes in open probability (P_o_) and unitary conductance [26], [27]. The KCNE1-Δ70 mutant yielded a similar current density reduction as that of the D76N. To determine if the KCNE1 mutants affected current density by altering the amount of KCNQ1 channel protein we examined immunoblots from cells expressing the various combinations of channel subunits (Figure 2C). Neither wild type KCNE1 nor its mutants changed the abundance of KCNQ1 protein. Immunoblots in Figure 2C show that comparable amounts of the different KCNE1 proteins are expressed but do not specifically address surface expression. That the current is transformed by both the KCNE1-D76N and KCNE1-Δ70 is solid evidence for surface localization and association with KCNQ1. A recent report suggests that KCNE1-D76N is shunted away from the plasma membrane by endocytosis without trafficking back via recycling endosomes when cells are stimulated with SGK1 [28]. Such a mechanism may play a role in the generally observed reduction of current density with the mutant subunit but in our studies there was no additional SGK1 stimulation which may explain the comparably abundant mutant and wild type protein.

**Figure 3:**
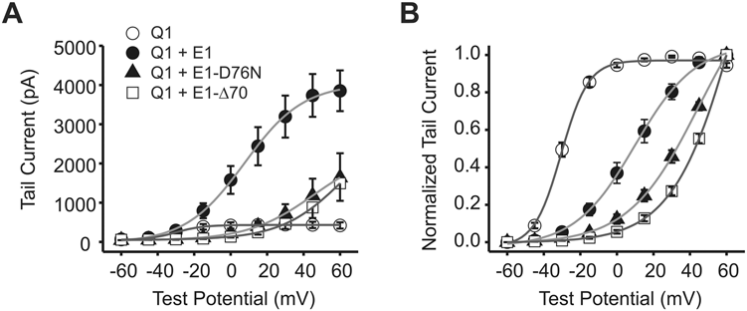
KCNE1-D76N and KCNE1-Δ70 shift the voltage dependence of activation of I_Ks_. A) voltage-dependent activation curves, and B) normalized activation curves. Curves were fitted using a Boltzmann function. V_h_ for KCNQ1 = −30.3±1.0 mV, KCNQ1/KCNE1 = 9.5±4.2 mV, KCNQ1/KCNE1-D76N = 43.5±3.0 mV, KCNQ1/KCNE1-Δ70 = 71.5±7.0 mV. n = 6, p<0.001 for both mutants compared to wild-type KCNE1.

The C-terminal KCNE1 mutations also caused a more pronounced rightward shift of the voltage-dependence of activation (VDA) when compared to wild type KCNE1 (Figure 3). The V_h_ values, as estimated by Boltzmann curve fits of VDA data, are listed in Table 1 (p<0.001 for both mutants compared to wild-type KCNE1). That the C-terminal mutants shifted the VDA suggests that the C-terminus contributes to activation gating mechanism by stabilizing the closed state or destabilizing the open state without affecting the kinetics of activation.

**Table 1:** Biophysical characteristics of KCNE1 mutants.

	Current Density	Activation Time ½ Max	V_h_	ΔG(C→O)	ΔE_a_(O→C)
Units	pA/pF	msec	mV	kJ/mol	kJ/mol
KCNQ1	35.22±8.00	22.28±1.40	−30.3±1.01	ND	ND
KCNQ1+KCNE1	410.92±38.39	1150.65±136.66	9.53±4.16	1.58	ND
KCNQ1+KCNE1-D76N	102.95±20.41	1338.88±59.42	43.46±2.97	5.75	−2.50±0.32
KCNQ1+KCNE1-Δ70	63.95±16.97	1209.37±62.63	71.74±7.04	9.8	−3.14±0.38

N = 6.

Abbreviations: V_h_ voltage at which half the channels are activated; ΔG(C→O) change in Gibbs free energy for the closed and open states during channel activation; ΔE_a_(O→C) change in activation energy for the transition from open to closed states relative to wild-type KCNE1. (ND, not done).

**Figure 4:**
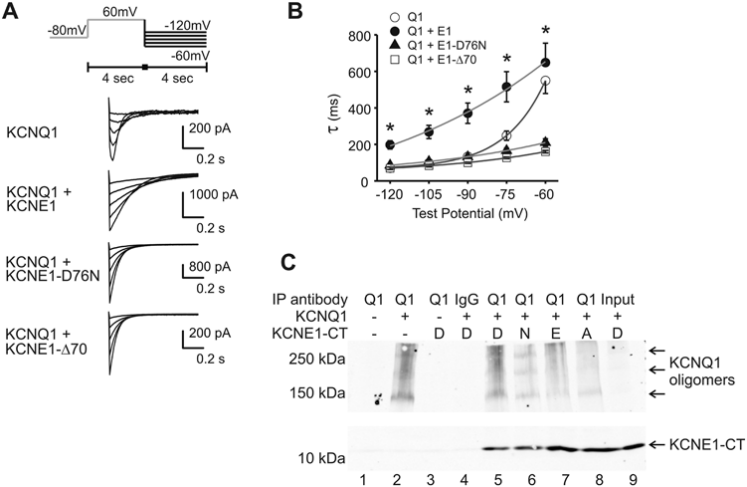
Deactivation rates are accelerated by both KCNE1-D76N and KCNE1-Δ70. A) Current traces, with deactivation protocol in inset, and B) rates of deactivation plotted against voltage from CHO cells transiently expressing KCNQ1 (Q1) either without KCNE1 (E1), or with wild-type E1, E1-D76N, or E1-Δ70. Asterisks (*) indicate significant change between wild-type E1 and both E1 mutations. n = 6. C) Co-immunoprecitation experiment of C-termini KCNE1 with full length KCNQ1. Full length KCNQ1 was expressed with KCNE1 C-terminus (KCNE1-CT), either wild-type or with D76E, D76N, or D76A point mutations. Immunoprecipitation was performed with goat anti-KCNQ1 antibody, and immunoblot with rabbit anti-FLAG antibody (Santa Cruz). For controls, lane 1 shows results from untransfected cells, lane 2 from KCNQ1 alone, lane 3 from KCNE1 wild type (D76) alone, and lane 4 from KCNQ1 and KCNE1 with control antibody. The different KCNE1 forms are designated by the single amino acid letter at the 76^th^ position.

Figure 4A illustrates the involvement of KCNE C-terminus in the kinetics of deactivation. Co-expression of KCNQ1 and KCNE1 results in slowed deactivation kinetics, as well as the removal of the inactivation “hook” on the tail current. Both KCNE1 mutations significantly accelerate deactivation at all voltages compared to wild type KCNE1. The mutants also accelerated deactivation compared to KCNQ1 alone at voltages positive to −80 mV (Figure 4B). These results suggest that the C-terminus of KCNE1 interacts with the channel to determine voltage-dependent deactivation. This regulation may occur through interactions between the KCNE1 terminus and intracellular portions of KCNQ1. Mutations in the KCNE1 C-terminus may thus affect the normal interaction between the proteins via disruption or the formation of new interactions that are consequential to deactivation rates. Though the deactivation profile of the KCNE1-D76N mutant resembles that of the KCNE1-Δ70 mutant, the D76N mutation did not abolish the interaction between the KCNQ1 and KCNE1 C-termini. Co-expression of KCNE1 C-terminal fragments (lacking the N-terminus and the transmembrane domain) with full-length KCNQ1 resulted in quantifiable binding (detected by co-immunoprecipitation) that was not prohibited by the KCNE1-D76N mutation nor other D76 mutations (D76E or D76A) (Figure 4C). Thus, any functional change caused by this mutation is not due to elimination of interaction between KCNQ1 and the C-terminus of KCNE1, but rather by more subtle changes in interaction that result in a change in channel gating.

**Figure 5:**
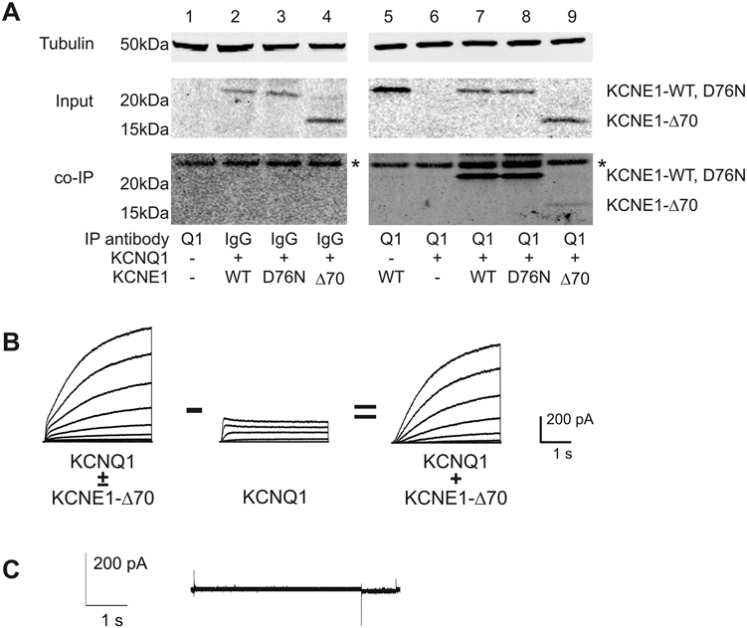
A) Co-immunoprecipitation of wild-type and mutant KCNE1 with KCNQ1. KCNQ1 (Q1) was expressed with KCNE1 (E1), E1-D76N, or E1-Δ70. KCNQ1 was immunoprecipitated with goat anti-KCNQ1 antibody (Santa Cruz), and western blot analysis performed with mouse anti-FLAG antibody (Sigma). The asterisks (*) indicate a row of nonspecific bands that appeared in every lane, including control lanes. For controls, lane 1 shows results from untransfected cells, lanes 2–4 from cells transfected with KCNQ1 and KCNE1 (WT, D76N, or Δ70 as indicated) and pulled down with control IgG. B) Digital subtraction of Q1 current from mixed Q1-E1 current (E1-Δ70 mutant). Left tracing shows the currents obtained from a cell transfected with a 1∶1 ratio of KCNQ1∶KCNE1-Δ70 plasmids. Middle tracing shows a pure KCNQ1 current scaled to match the amplitude of the initial rapid current deflection seen in the mixed current to the left. Right side tracing shows the resulting digital subtraction of the two currents resulting in a current with slow sigmoidal activation characteristic of I_Ks_. C) Voltage clamp tracing from cells transfected with KCNE1-Δ70 plasmid alone demonstrating that the mutant KCNE1 did not induce any other conductances on its own.

We also investigated the physical association of KCNE1 C-terminal mutants with KCNQ1 channels by co-immunoprecipitation (co-IP) experiments. As shown in Figure 5A, KCNE1-D76N and KCNE1-Δ70 achieve expression levels as high as or higher than wild type KCNE1 in CHO cells co-transfected with KCNQ1. When KCNQ1 was immunoprecipitated, wild type and D76N KCNE1 co-precipitated to a comparable extent. The KCNE-Δ70 mutant, however, co-precipitated to a lesser degree suggesting that the truncated mutant had an intrinsically lower affinity for the channel, while the wild type and D76N mutant had similar affinity or association. At this point however, we cannot exclude the possibility that the KCNE1-D70 binds to another cellular protein with higher affinity than KCNQ1, thereby preventing it from participating in channel formation. The lower apparent affinity of the Δ70 mutant was also functionally manifest as current with activation kinetics different than either KCNQ1 alone or KCNQ1/KCNE1 when cells were transfected with equimolar amounts of KCNQ1 and KCNE1-Δ70 (Figure 5B). These hybrid currents had initial fast activation characteristic of KCNQ1, followed by slow continued activation characteristic of I_Ks_. When a pure KCNQ1 current was scaled to the amplitude of the initial rapid activation phase of the hybrid current and digitally subtracted, the resulting signal recapitulated I_Ks_ activation kinetics of KCNQ1/KCNE1. To ensure that the KCNE1-Δ70 protein did not generate any unpredicted conductances that might contaminate the KCNQ1/KCNE1 channel currents, we examined cells transfected with the KCNE1-Δ70 alone and observed no currents (Figure 5C). Thus, the hybrid current represented an algebraic sum of two populations of channels—KCNQ1 alone plus KCNQ1/KCNE1-Δ70. To achieve saturation of KCNQ1 with KCNE1-Δ70 subunits we transfected cells with higher KCNE1 to KCNQ1 cDNA ratios. Cells showing mixed currents due to non-stoichiometric association were not included in the kinetic analyses. In this way we were able to distinguish between association/assembly functions of the KCNE1-C-terminus and the purely biophysical functions. That KCNE1-Δ70 truncation results in lower amounts co-immunoprecipitated by KCNQ1, suggests a possible role for the KCNE1 C-terminus that favors association or assembly of the channel complex but is not absolutely required.

**Figure 6:**
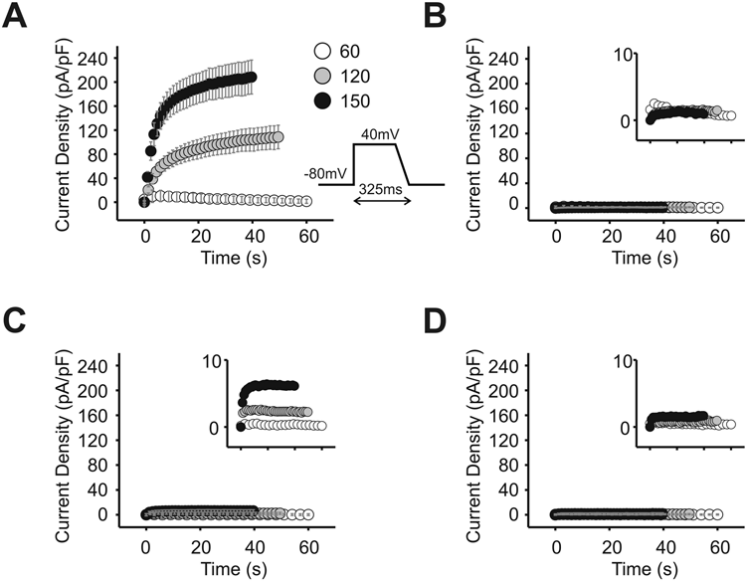
KCNE1-D76N and KCNE1-Δ70 mutations result in defective rate-related accumulation of I_Ks_ current. A series of 100 pulses mimicked action potential trains at three rates: 60, 120, or 150 per minute. On the voltage protocol, the grey stripe indicates the 10 ms over which the current measured was averaged and plotted on the graphs. The insets for B, C, and D show the Y-axis magnified and the points plotted without error bars. X- and Y-axes labels are the same as the main graph. A) Cells transfected with KCNQ1 (Q1) and wild-type KCNE1 (E1); depolarization length 325 ms. B) Cells transfected as in A, but depolarization length of voltage protocol adjusted as follows: 60 pulses/min-325 ms; 120 pulses/min-175 ms; 150 pulses/min-145 ms. C) Cells transfected with Q1 and E1-D76N; depolarization length 325 ms. D) Cells transfected with Q1 and E1-Δ70; depolarization length 325 ms. n = 6.

The physiological effects of C-terminal mutations were examined by assessing rate-related current accumulation. In normal cardiac physiology, action potential durations (APD) shorten in response to elevated heart rate, which is a necessary accommodation in order to maintain a proper ratio of time spent in electrical systole to time spent in diastole [29]–[33]. The inability to accommodate action potential duration results in early after-depolarizations, which can trigger tachyarrhythmias. The activity of delayed rectifier currents (I_Kr_ and I_Ks_) may contribute to the shortening of APDs through cycle-length-related accumulation of open channels [34]–[37]. In our whole-cell patch clamp recordings where we simulated trains of action potentials at different frequencies mimicking normal and elevated heart rates, wild-type KCNQ1/KCNE1 channels exhibited marked accumulation of K^+^ conductance at the frequencies between 120 and 150 depolarizations per minute (Figure 6A). As expected, when the length of depolarization was shortened proportionally to cycle length (i.e. shortened APD for faster rates), accumulation did not occur (Figure 6B). Neither KCNQ1/KCNE1-D76N nor KCNQ1/KCNE1-Δ70 exhibited accumulation of current (Figures 6C and 6D). This defective current accumulation at shorter cycle lengths may contribute to triggering arrhythmias in patients with LQTS mutations that affect C-terminal interactions between KCNQ1 and KCNE1.

## Discussion

The results presented here provide evidence that the KCNE1 C-terminus plays an important role in the regulation of KCNE1 channels, and in I_Ks_ adaptation to changes in heart rate. The C-terminus of KCNE1 is necessary to transform the fast deactivation kinetics of KCNQ1 current to the slow deactivation kinetics of I_Ks_. Furthermore, the deactivation kinetics of I_Ks_ are sensitive to an LQT-linked single amino acid mutation in the C-terminus of KCNE1. The presence or absence of the C-terminus of KCNE1 plays a role in the affinity or likelihood of association of KCNE1 with KCNQ1. The KCNE1-D76N mutation however, did not exert its effect via disruption of association. The acceleration of channel deactivation by KCNE1 C-terminal point mutation or truncation also renders the channel incapable of adapting to increases in heart rate, which may contribute to some LQT5 phenotypes.

Previous studies have identified the precise site in the transmembrane domain of KCNE1 that controls I_Ks_ activation kinetics [11], [12]. These studies, however, also showed that deactivation kinetics are not controlled by the same region [11]. Evidence from several groups suggests the importance of the C-terminus as a region critical for normal KCNE1 function. Early on, Takumi et al. showed that mutations in the C-terminal region of KCNE1 were poorly tolerated, resulting in a reduction in channel activity [38]. The effects of several of these mutations, including D76N, were confirmed by Sesti et al., who showed that the mutant channels had lower unitary currents and diminished open probabilities, but only minor changes in ionic permeability [27].

Our present data confirms that control of I_Ks_ activation rate lies outside of the C-terminus: the KCNE1-Δ70 truncation mutation did not alter the activation profile of I_Ks_ when coupled to KCNQ1. Neither KCNE1-D76N or -Δ70 produced a channel with KCNQ1 that yielded constitutively activated conductance as has been seen with KCNE3 [11], [12], [39]. The voltage dependence of activation, however, was significantly right-shifted by ∼60 mV. A shift in VDA indicates a change in the relative free energy (ΔG) of the closed and open states of the channel, versus a change in the activation energy (or transition-state energy barrier, ΔE_a_), which would be reflected by altered activation kinetics. The other prominent effect of the KCNE1-Δ70 mutation was the significant acceleration of deactivation kinetics of the KCNQ1/KCNE1 channel.

Similar results characterize the functional effects of the D76N mutant. The increase in the ΔG of opening of both mutants compared to wild-type causes a rightward shift in the VDA curve that indicates either a stabilization of the closed state or destabilization of the open state. There was little difference in activation energy for channel opening with the KCNE1 mutants since the kinetics for channel activation were largely unchanged (Table 1). There were however, substantial differences in the calculated change in activation energy for channel deactivation (ΔE_a_(O→C)) that are compatible with a model wherein the mutant subunits have a greater effect on the channel's open state than the closed state. The fact that ΔE_a_(O→C) is affected by the same mutations that affect the ΔG of the open state suggests that the transition state is similar to the open state, and hence one of the earliest structural changes to occur during channel deactivation is a conformational change at the C-terminus of KCNE1 that results in channel closure. Taken together, these data suggest that the C-terminus of KCNE1 plays a role in determining the relative free energy of the open and closed states of the channel, without affecting the activation energy required to traverse from closed to open. Furthermore, the results suggest that the wild type KCNE1 C-terminus interaction affects the transition-state energy barrier of the channel primarily during the open-to-closed transition, thereby slowing the deactivation rate. Thus, the absence of the C-terminus appears to affect the relative free energy state in favor of a stabilized closed state, and lowers the energy barrier for the open-to-closed transition.

The D76N mutation was one of the first two (along with S74L) mutations in KCNE1 that were associated with LQTS, providing evidence that KCNE1 presented a separate LQTS locus, LQT5 [15], [40]. It is interesting then that this single amino acid change, D76N, produces such similar results as the Δ70 truncation—significantly faster deactivation rates and ∼35 mV right shift in VDA. The similarities are not due to an abolishment of interaction between the KCNE1 C-terminus and KCNQ1 by the D76N mutation. When just the C-terminal portion of KCNE1-D76N is expressed with full-length KCNQ1, quantifiable binding occurs as detected by immunoprecipitation and immunoblot. The change from an acidic R-group to a polar, uncharged R-group, however, may significantly affect how the residue interacts with its environment, yielding a C-terminus that cannot properly stabilize the open state of the KCNQ1/KCNE1 channel or slow its deactivation kinetics.

Furthermore, our data provide evidence for a role for the KCNE1 C-terminus in KCNQ1/KCNE1 channel assembly. The D76N mutation does not reduce the total KCNE1 protein that can be co-immunoprecipitated with KCNQ1, but the Δ70 truncation significantly reduces co-immunoprecipitated KCNE1. The reduced likelihood of assembly of the KCNE1-Δ70 mutant with KCNQ1 was also demonstrated by the difficulty in achieving pure I_Ks_ current when co-transfecting equimolar KCNQ1 and KCNE1-Δ70. Pure saturated KCNQ1/KCNE1-Δ70 currents were only reliably achieved by increasing the ratio of KCNQ1-Δ70:KCNQ1 cDNA. Thus, C-terminal KCNE1 mutations may impair the normal formation of KCNQ1/KCNE1 channels that are successfully trafficked to the plasma membrane. In our functional studies, where we were able to saturate the KCNQ1 channels with KCNE-Δ70 subunits, thereby producing current with I_Ks_-like activation kinetics, the overall current density was reduced. This may be due to an overall reduction in surface expression of KCNQ1/KCNE1-Δ70 channels, because achieving a workable ratio of KCNQ1 to KCNE1-Δ70 DNA necessitated reducing the amount of KCNQ1 DNA used in the transfections.

Because we were able to achieve saturated I_Ks_-like current regardless of the imposed mutations, our electrophysiological studies of current accumulation under increasing depolarization frequency reflect the cumulative effects of changes in deactivation kinetics, VDA, and current density. In normal cardiac physiology, action potential durations (APD) shorten to accommodate for increases in heart rate [29]–[33]. As cycle-length decreases, KCNQ1/KCNE1 channels accumulate in the open state, resulting in an increased potassium conductance and shortened repolarization phase after subsequent depolarizations. This accommodation is necessary to maintain the proper electrical systole∶diastole duration ratio. A failure to properly rate-accommodate can lead to tachyarrhythmias. In our current accumulation protocol, the normal accumulation of open wild-type KCNQ1/KCNE1 channels occurs during increased depolarization frequency without adjusting the duration of the depolarization. With each depolarization, there is a steady increase in current density (Figure 6A). If the action potential duration is reduced in proportion to the frequency, the current accumulation is completely abolished because there is adequate time for the channels to deactivate (Figure 6B). When we use the fixed-cycle length protocol (as in 6A) with either the D76N or Δ70 mutations, no current accumulation occurs. We hypothesize that a combination of increased deactivation rates and the positive shift in VDA contribute to the defective current accumulation.

Taken together, these findings of channel kinetics and subunit association we can now firmly delineate the relative contributions of KCNE1 C-terminus and TMD. The kinetics of activation are clearly controlled by the TMD, thereby obviating the need for a complex bipartite model previously advocated [39]. Our data reveal several possible roles for the KCNE1 C-terminus in regulating KCNQ1/KCNE1 channel assembly and I_Ks_ current. Thus, there may be multiple mechanisms in which mutations in the C-terminus cause the exercise-induced phenotypes of LQT1 and LQT5. Mutations may reduce affinity of KCNE1 for KCNQ1, thereby decreasing the overall surface expression of functional channels. Other mutations may allow normal assembly, but affect the thermodynamics or kinetics of gating resulting in impaired ability to adapt to changes in heart rate. The significance of the KCNQ1 and KCNE1 C-terminal regions warrants more detailed studies into how they interact structurally, and how disease-causing mutations compromise these interactions.

## Methods

### Plasmids and Cell Culture

Construction and validation of Myc-KCNQ1 and 3X-FLAG-KCNE1 expression plasmids have been previously described [13], [41]. KCNE1-D76N and KCNE1-Δ70 mutations were introduced by QuikChange® Site-Directed mutagenesis (Stratagene). Primers used for mutagenesis were as follows: KCNE1-D76N (5′-CAC TCG AAC ***A***AC CCA TTC AAC-3′); and KCNE1-Δ70 (5′- AGC TAC ATC CGC TCC AAG ***T***AG-3′). KCNE1 C-terminus was cloned from the expression plasmid with the following primers: 5′-CGG AAT TCC ATG CGC TCC AAG AAG CTG GAG CAC-3′ and 5′-CGG GAT CCT CAT GGG GAA GGC TTC GTC TC-3′. Subsequently, mutagenesis was performed to introduce the D76A, D76E, and D76N mutations. Primers used for mutagenesis were as follows: D76A (5′-CAC TCG AAC G**C**C CCA TTC AAC-3′), D76E (5′-CAC TCG AAC GA**A** CCA TTC AAC-3′), D76N (5′-CAC TCG AAC **A**AC CCA TTC AAC-3′). Sequences of mutated cDNAs were verified by automated sequencing. Chinese Hamster Ovary (CHO) cells (American Type Culture Collection) were maintained in Ham's F-12 (Mediatech Inc.) supplemented with 10% FBS (Hyclone) and Penicillin/Streptomycin (Mediatech Inc.) at 37°C and 5% CO_2_. Transient transfections were performed using Lipofectamine 2000 (Invitrogen). Molar ratios of 7∶7∶2 of KCNQ1∶KCNE∶GFP plasmid were used to allow identification of transfected cells by fluorescence. Electrophysiology studies were performed 24–48 hr after transfection.

### Electrophysiology

Transfected CHO cells were grown on sterile glass coverslips and placed in an acrylic/polystyrene perfusion chamber (Warner Instruments) mounted in an inverted microscope outfitted with fluorescence optics and patch pipette micromanipulators. Extracellular solution consisted of NaCl 150 mM, CaCl_2_ 1.8 mM, KCl 4 mM, MgCl_2_ 1 mM, glucose 5 mM, and HEPES buffer 10 mM (pH 7.4) at room temperature (20–22°C). Intracellular pipette solution contained KCl 126 mM, K-ATP 4 mM, MgSO_4_ 1 mM, EGTA 5 mM, CaCl_2_ 0.5 mM, and HEPES buffer 25 mM (pH 7.2) at room temperature. The whole-cell configuration of the patch clamp technique was used to measure potassium currents (Hammill et al., 1981). A MultiClamp 700B patch-clamp amplifier was used and protocols were controlled via PC using pCLAMP10 acquisition and analysis software (Axon Instruments). Patch clamp pipettes were manufactured and tips polished to obtain a resistance of 2–3 megaOhm in the test solutions. The pipette offset potential in these solutions was corrected to zero just prior to seal formation. The junction potential for these solutions was calculated between 3–4 mV (by pClamp analysis software) and was not corrected for analyses. Whole cell capacitance (generally 10–25pF) was compensated electronically through the amplifier. Whole cell series resistance of 6–12 MΩ was compensated to 75–90% using amplifier circuitry such that the voltage errors for currents of 2nA were always less than 6 mV. A standard holding potential was −80 mV, and figure insets show applied voltage protocols. Data was filtered using an 8-pole Bessel filter at 1 kHz. Activation kinetics were quantified as a rise time to half max following a 60 mV depolarizing step from −80 mV holding potential. Deactivation kinetics were well-fit using a single standard exponential. For the K^+^ conductance accumulation protocol analysis, outward current during the first 10 ms of the beginning of each depolarization was averaged.

### Co-immunoprecipitation

CHO cells in 60 mm dishes 36–48 hours post-transfection were placed on ice and lysed with ice-cold NDET buffer (150 mM NaCl; 1% NP-40; 0.4% Deoxycholic acid; 5 mM EDTA; and 25 mM Tris (pH 7.4)) with complete protease inhibitor cocktail (Roche Applied Science, EDTA-free) for 15 minutes. Cells were then scraped into 1.5 mL tubes, incubated on ice for 30 minutes, and then centrifuged at 16,000×g for 10 minutes at 4°C to remove nuclei and cellular debris. The supernatant was pre-cleared with 20 µL normal goat IgG (Santa Cruz) and 20 µL Immunopure Ultralink protein G agarose beads (Pierce) for 1 hour at 4°C. After centrifuging at 5,500×g for 1 minute at 4°C, the supernatant was incubated with 40 µL goat-anti-KCNQ1 antibody (Santa Cruz) and 30 µL protein G beads overnight at 4°C. The beads were then washed four times with ice-cold NDET, and eluted by incubation with 4× SDS-PAGE sample buffer (4% SDS (w/v), 40% glycerol, 20% β-mercaptoethanol (v/v), 0.004% bromphenol blue (w/v), and 125 mM Tris·HCl, pH 6.8) for 30 min at room temperature prior to SDS-PAGE analysis.

### SDS-PAGE and Western Blotting

Immunoprecipitated samples were separated on 4–20% gels (BioRad Criterion™), and transferred onto nitrocellulose membrane (BioRad) by semi-dry blotting unit (FisherBiotech). Membranes were blocked with 5% nonfat dry milk in Tris-buffered saline (TBS) for 30 minutes at room temperature, and then incubated with anti-*myc* (1∶250, Santa Cruz) and anti-FLAG (1∶5000, Sigma) antibody in 5% non-fat dry milk and 0.05% Tween 20 in TBS for 2 hours at room temperature or overnight at 4°C. The membranes were then washed four times for 5 minutes each with 0.05% Tween 20 in TBS and incubated in the corresponding infrared-fluorescence IRDye® 800 conjugated donkey anti-mouse and IRDye® 700DX conjugated donkey anti-goat secondary antibodies (Rockland Immunochemicals Inc.) diluted 1∶10,000 for 30 minutes at room temperature in the dark followed by washing with 0.05% Tween 20 in TBS. The membranes were then scanned to visualize the signal at 680 nm or 780 nm by the Odyssey detection system (Li-Cor Biosciences).

### Linear Free Energy Relationship Calculations

Values for the change in Gibb's free energy (**Δ** ***G***) were calculated from the isochronal tail currents of each ion channel complex. The tail currents were fit to a Boltzmann sigmoid curve with equation:
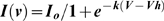
Where
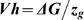
and
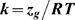
In this equation, ***V_h_*** represents the voltage at which ½ of the maximal activation occurs and ***k*** is the slope factor. The ***V_h_*** and slope factor (now termed ***SF***) are related to the Δ***G*** by the equation:
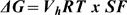

***E_a_***, the activation energy for the transition from the open to closed state is related to the first order rate constant for channel deactivation by the equation:
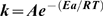
Where ***k*** is the 1^st^-order rate constant of the reaction and ***Ea*** is in J/mol. Given a wild-type and mutant channel, by taking the ratio of their first order rate constants we can approximate the change in activation energy (assuming that the proportionality constant ***A*** does not change), as shown below: